# 

**Published:** 1865-08

**Authors:** 


### CONTENTS.

### ORIGINAL CONTRIBUTIONS.

ART. XXVII. REPORT ON ORTHOPEDIC SURGERY, FOR THE ANNUAL MEETING OF THE ILLINOIS STATE MEDICAL SOCIETY, HELD IN BLOOMINGTON, MAY 2d, 1865. By DAVID PRINCE, M.D., of Jacksonville, Illinois. With 23 Illustrations, ........ 449

### EDITORIAL.

Explanation, ........ 532
Surgeons for the Army, ........ 532
Immoral Medical Advertisements, ........ 532
Proceedings of the Fox River Valley Medical Association, ........ 533
Letter from Prof. Johnson, in Paris, ........ 535
Expulsion of a Fleshy Mass from the Uterus. By John D. Jennings, Sonora, Ohio, ........ 541

